# Urotensin II Inhibits Doxorubicin-Induced Human Umbilical Vein Endothelial Cell Death by Modulating ATF Expression and via the ERK and Akt Pathway

**DOI:** 10.1371/journal.pone.0106812

**Published:** 2014-09-30

**Authors:** Yen-Ling Chen, Yi-Ting Tsai, Chung-Yi Lee, Chien-Hsing Lee, Chung-Yi Chen, Chi-Ming Liu, Jin-Jer Chen, Shih-Hurng Loh, Chien-Sung Tsai

**Affiliations:** 1 Graduate Institute of Medical Sciences, National Defense Medical Center, Taipei, Taiwan, Republic of China; 2 Department of Nursing, Min-Hwei College of Health Care Management, Tainan, Taiwan, Republic of China; 3 School of Medical and Health Sciences, Fooyin University, Kaohsiung, Taiwan, Republic of China; 4 Division of Cardiology, Department of Internal Medicine and Graduate Institute of Clinical Medical Science, China Medical University, Taichung, Taiwan, Republic of China; 5 Institute of Biomedical Sciences, Academia Sinica, Taipei, Taiwan, Republic of China; 6 Division of Cardiology, Department of Internal Medicine, China Medical University Hospital, Taichung, Taiwan, Republic of China; 7 Department of Pharmacology, National Defense Medical Center, Taipei, Taiwan, Republic of China; 8 Division of Cardiovascular Surgery, Department of Surgery, Tri-Service General Hospital, National Defense Medical Center, Taipei, Taiwan, Republic of China; University of Sassari, Italy

## Abstract

**Background and Purpose:**

Regulation of the homeostasis of vascular endothelium is critical for the processes of vascular remodeling and angiogenesis under physiological and pathological conditions. Urotensin II (U-II), a potent vasoactive peptide, participates in vascular and myocardial remodeling after injury. We investigated the protective effect of U-II on doxorubicin (DOX)-induced apoptosis in cultured human umbilical vein endothelial cells (HUVECs) and the potential mechanisms involved in this process.

**Experimental Approach:**

Cultured HUVECs were treated with vehicle, DOX (1 µM), U-II, or U-II plus DOX. Apoptosis was evaluated by DNA strand break level with TdT-mediated dUTP nick-end labeling (TUNEL) staining. Western blot analysis was employed to determine the related protein expression and flow cytometry assay was used to determine the TUNEL positive cells.

**Key Results:**

U-II reduced the quantity of cleaved caspase-3 and cytosol cytochrome c and increased Bcl-2 expression, which results in protecting HUVECs from DOX-induced apoptosis. U-II induced Activating transcription factor 3 (ATF3) at both mRNA and protein levels in U-II-treated cells. Knockdown of ATF3 with ATF3 siRNA significantly reduced ATF3 protein levels and U-II protective effect under DOX-treated condition. U-II downregulated p53 expression in DOX-induced HUVECs apoptosis, and it rapidly activated extracellular signal-regulated protein kinase (ERK) and Akt. The DOX induced change of p53 was not affected by U-II antagonist (urantide) under ATF-3 knockdown. The inhibitory effect of U-II on DOX-increased apoptosis was attenuated by inhibitors of ERK (U0126) and PI3K/Akt (LY294002).

**Conclusion and Implications:**

Our observations provide evidence that U-II protects HUVECs from DOX-induced apoptosis. ERK-Akt phosphorylation, ATF3 activation, and p53 downregulation may play a signal-transduction role in this process.

## Introduction

Vascular endothelial cell injury is the critical event in the pathogenesis of cardiovascular diseases [1]. Prevention of vascular endothelial cell apoptosis may ameliorate endothelial function and angiogenesis [2]. Therefore, anti-apoptotic agents may be potential candidates that affect vascular remodeling, which is known to be a key mechanism in the progression of atherosclerosis and other cardiovascular diseases.

One of the most potent vasoactive peptides is urotensin-II (U-II; also known as urotensin-2), which is a cyclic peptide synthesized through proteolytic cleavage of a precursor molecule, prepro-U-II [3]. U-II signaling has been identified to be via the urotensin receptor (previously called GPR14) [4]. U-II and GPR14 are highly expressed in endothelial and smooth muscle cells involved in vascular remodeling [5]. They have been associated with several cardiovascular pathologies including pulmonary vascular and atherosclerosis remodeling [5], [6]. Our previous study [2] also validated U-II plays an important role in cardiovascular remodeling. However, the molecular mechanisms underlying activation of endothelial cells by U-II are still unclear.

Doxorubicin (DOX) is a well-established and a highly effective anti-neoplastic agent [7]. However, limitations of the clinical use of DOX are its severe side effects, including cardiotoxicity and nephrotoxicity [8]. Apoptotic cell death has been reported to be a key component in DOX-induced cardiotoxicity [9], [10]. Furthermore, DOX induces caspase-dependent apoptotic signaling in endothelial cells [11]. Pro-apoptotic proteins such as Fas, anti-apoptotic proteins such as Bcl-2, the tumor suppressor protein p53, and the PI3K/Akt pathway are involved in DOX -induced apoptosis in human umbilical vein endothelial cells (HUVECs) [12], [13]. However, U-II treatment to protect vascular endothelial cells from being affected by DOX has not been explored. We accordingly investigated the effect of U-II on DOX-induced apoptosis in HUVECs and on the related signaling pathways.

## Methods

### Reagents

Dulbecco's modified Eagle's medium (DMEM), fetal calf serum, and tissue culture reagents were purchased from Invitrogen Corporation (Carlsbad, CA, USA). U-II and all other chemicals of reagent grade were obtained from Sigma-Aldrich Chemical Co. (St. Louis, MO, USA). Urantide was obtained from Peptide International (Louisville, Kentucky, USA). Antibodies were purchased from Lab Frontier Co. Ltd., Seoul, Korea (anti-GAPDH), and Cell Signaling Technology, Inc., Danvers, MA, USA (anti-caspase-3, anti-phospho-specific, PARP, p53, ATF3 and total Akt, ERK).

### Endothelial cell culture and treatments

HUVECs were obtained from PromoCell (Heidelberg, Germany) as cryopreserved cells. After thawing, cells were plated in cultured flasks and cultured to confluence in MCBD 131 medium (PromoCell) containing 28 mM hydroxyethylpiperazine ethanesulfonic acid, 2% fetal calf serum, 0.1 ng ml human recombinant epidermal growth factor, 1 ng ml human recombinant basic fibroblast growth factor, 50 µg ml gentamycin, 50 ng ml amphotericin B, and 1 µg ml synthetic hydrocortisone and supplemented with a mixture (PromoCell) containing endothelial cell growth factor and heparin. Cells were grown at 37°C in a humidified 5% CO_2_ atmosphere for 3–4 days. Confluent cultures between passages 2 and 10 were used for all experiments. Cells were cultured in serum-free medium for 24 h prior to addition of 1 µM DOX in fresh serum-free medium for 24 h. U-II was added at indicated concentrations 24 h prior to DOX treatment. In experiments involving kinase inhibitors, cells were cultured in serum-free-medium for 24 h, after which the inhibitors LY294002 (20 µM), U0126 (10 µM) or urantide (10 µM) were added 0.5 h prior to U-II and/or DOX treatments. All inhibitors were freshly constituted in DMSO and protected from light.

### TUNEL assay

DOX -mediated apoptosis in HUVECs was detected with enzymatic labeling of DNA strand breaks, which were identified using terminal deoxynucleotidyl transferase-mediated deoxyuridine triphosphate nick end-labeling (TUNEL) stain with a Cell Death Detection kit (Roche, Mannheim, Germany) according to the manufacturer's instructions. The apoptotic ratio was measured by flow cytometry according to the manufacturer's instructions.

### Caspase-3 activity assay

In the present caspase-3 activity assay, the caspase-3 substrate rhodamine-110 (Z-DEVD-R110) was used as a pre-fluorescent substrate. Activity of caspase-3 was determined using a commercial kit (Promega; Madison, WI, USA) according to the manufacturer's instructions. Briefly, after 24 h treatments with DOX, U-II, DOX plus U-II, or vehicle, caspase-3 reagent was added and incubated for 10 h. Levels of release of rhodamine-110 were measured with a luminescence spectrometer LS55 (Perkin-Elmer) at an excitation wavelength of 499 nm and an emission wavelength of 521 nm.

### Western blot analysis

Western blot analysis was performed as previously described [14]. Membranes were blocked in 10 mM Tris (pH 7.5), 100 mM NaCl, and 0.1% Tween 20 containing 5% nonfat dry milk, followed by incubation with primary antibody. Membranes were washed three times and incubated with the appropriate horseradish peroxidase-conjugated secondary antibody (1∶5000 dilutions) to detect bands by enhanced chemiluminescence (Amersham Biosciences Corp, NJ, USA).

### MTT assay

HUVECs viability was determined by using 3-(4,5-dimethylthiazol-2-yl)- 2,5-diphenyl tetrazolium (MTT, Sigma-Aldrich). In brief, cells were seeded with culture medium in 96-well microplates (4500 cells/well) and incubated at 37°C for 24 h before drugs exposures. At the end of treatments with drugs, cells were incubated with completed-medium containing 0.4 mg/ml MTT at 37°C for 4 h. The reduced MTT crystals were dissolved in DMSO and the absorbance was detected at 570 nm with a plate reader.

### LDH assay

Lactate dehydrogenase release into culture medium (LDH assay) were used as an estimative of cell viability [15]. In brief, cells were seeded with culture medium in 96-well microplates (4500 cells/well) and incubated at 37°C for 24 h before drugs exposures. At the end of incubations, LDH assays were performed. Lactate dehydrogenase (LDH) activity in the culture medium was determined in agreement with manufacturer instructions (CytoTox 96-NonRadioactive Cytotoxicity Assay, Promega).

### RNA Isolation and Reverse Transcription–Polymerase Chain Reaction (RT-PCR)

Total RNA was extracted from HUVECs using the TRIzol method according to the protocol recommended by the manufacturer (Invitrogen, Carlsbad, CA, USA). For the RT-PCR analysis, 1 g of total RNA was reverse-transcribed into complementary cDNA using a Super Script II First-Strand-Synthesis System (Invitrogen Co., Carlsbad, USA). First-Strand cDNA was amplified by PCR using Taq DNA polymerase. The following protocol was used for each PCR: 94°C for 1 min (1 cycle), followed by 9°C for 1 min, 57°C for 1 min, and 72°C for 2 min (26 cycles), and a final extension phase at 72°C for 5 min. Primer sequences for human ATF-3 were 5′-CGGTTTCCGTCTGGGCTTCT-3′ (forward) and 5′-GCACCTCAAAGCTGTTCCGTCCC-3′ (reverse) [16]; for human p53 were 5′-CGGTTTCCGTCTGGGCTTCT-3′ (forward) and 5′-GCACCTCAAAGCTGTTCCGTCCC-3′ (reverse); for human GAPDH mRNA were 5′- TGAAGGTCGGTGTGAACGGATTTGG-3′ (forward) and 5′-ACGACATACTCAGCACCGGCCTCAC-3′ (reverse). PCR products were run on a 2% agarose gel containing 0.5 g/mL ethidium bromide. The expression of p53 (or ATF-3) was normalized to the expression levels of GAPDH.

### Flow cytometry

HUVECs were grown on 0.1% gelatin coated 6-well plates to confluence. HUVECs were stained with annexin V-FITC apoptosis detection kit (BioVision, CA, USA), and apoptotic cells identified and quantified by flow cytometry. Briefly, after exposing to different treatments, cells were washed with PBS and then harvested by trypsin-EDTA solution (Invitrogen). The cell suspensions were centrifuged at 1000 rpm for 5 min to remove trypsin-EDTA solution. Then the cells were re-suspended in 1X binding buffer and incubated with propidium iodide (PI), annexin V-FITC for 5 min at room temperature in the dark. The stained cells were analyzed on a FACS flow cytometry (FACS-SCAN, Becton- Dickinson, Franklin Lakes, NJ, USA.).

### Short interfering RNA (siRNA) transfection

ATF3 siRNAs were purchased from Santa Cruz Biotechnology. ATF3 siRNAs and mock control oligonucleotides were transfected using the Lipofectamine (Invitrogen) reagent according to the manufacturer's instructions. The final concentration of the ATF3 siRNAs for transfection was 100 nmol/L. Transfected cells were washed with PBS, and then incubated in new culture media for an additional 24 h for U-II and Western blot assays.

### Statistical analysis

Results are expressed as mean ± S.E.M. Statistical analysis was performed using Student's t-test or analysis of variance (ANOVA) using Prism version 3.00 for Windows (GraphPad Software, San Diego, CA, USA). A value of *P* <0.05 was considered to be significant.

## Results

### Effects of U-II on DOX-induced HUVEC cytotoxicity

**Figure 1:**
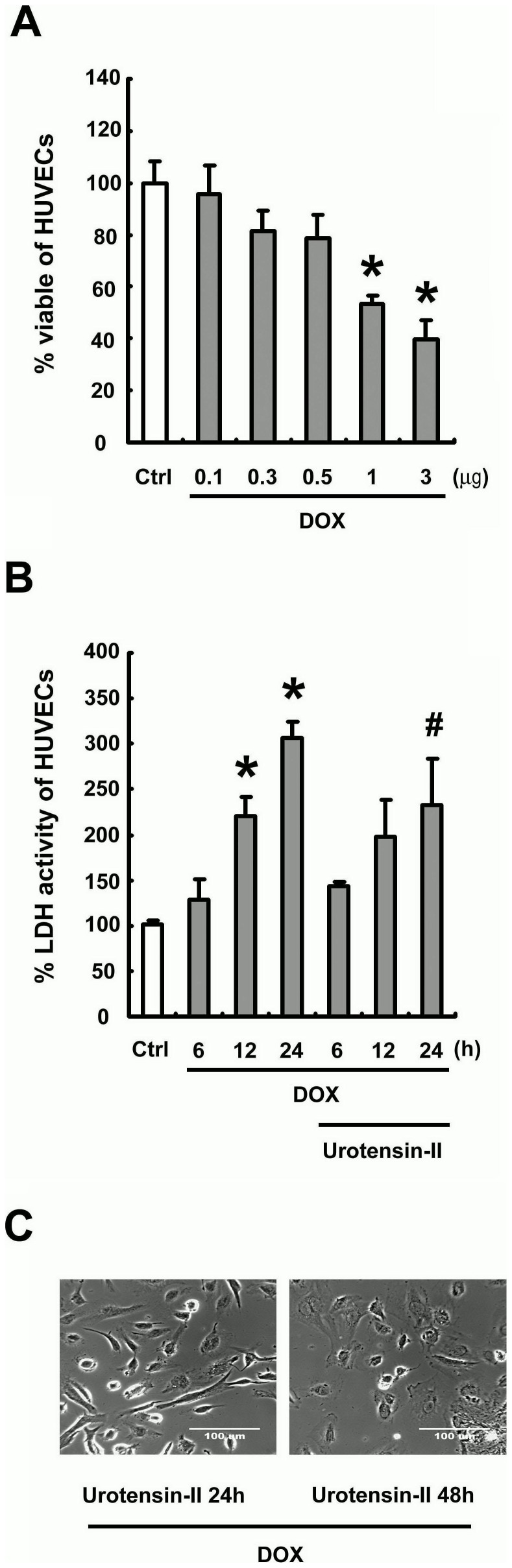
Effect of U-II on DOX-induced cytotoxicity evaluated by 3-[4,5-dimethylthiazol-2-yl]-2,5-diphenyl tetrazolium bromide (MTT) assay (see Methods) (A) 24 h incubation with DOX ranging from 0.1 to 3 µM inhibited the viability of HUVECs in a dose-dependent manner. The average of optical densities at 584 nm in control wells is expressed as 100%. (B) U-II inhibited LDH release from the cells by DOX after 24 h. (C) Photomicrographs from phase-contrast microscopy. Pretreatment with U-II attenuated the cytotoxic effect of DOX when cells were incubated with DOX for 24 h (left), however, the induction of cell death at 48 hr (right). Densitometric data are means ± SEM from three separated experiments. *P <0.05 vs. control (Ctrl); ^#^ *P* <0.05 vs. DOX treatment.

It has found that low concentrations of DOX, such as 1 µM, induced cell apoptosis in HUVECs [17]. To check cell toxicity caused by DOX in our cultured HUVECs, we have done the concentration-dependent testing. As shown in Figure 1A, 24 h incubation with DOX ranging from 0.1 to 3 µM inhibited the viability of HUVECs in a dose-dependent manner. However, no dose of U-II inhibited the decrease of MTT activity induced by further 24 h incubation with DOX (total, 48 h; data not shown). In contrast, LDH released from HUVECs increased after treatment with DOX. LDH release from HUVECs markedly increased after 12 h of incubation with 1 µM DOX. At the same time point, pretreatment with U-II inhibited LDH release from the cells by DOX. After 24 h incubation with DOX, LDH release increased approximately threefold compared with the control and increased approximately one-fold even with existing U-II (Fig. 1B). Fig. 1C shows morphological changes in HUVECs induced by 1 µM of DOX and the effect of 10 nM of U-II on DOX -induced cell toxicity. Pretreatment with U-II attenuated the cytotoxic effect of DOX when cells were incubated with DOX for 24 h. However, many vacuoles were formed in the HUVECs by DOX treatment despite the existing U-II (Fig. 1C, left). Further 24 h incubation (total, 48 h) of the cells with DOX eventually caused cell death, even with existing U-II (Fig. 1C, right).

### Effects of U-II on DOX -induced HUVEC apoptosis

**Figure 2:**
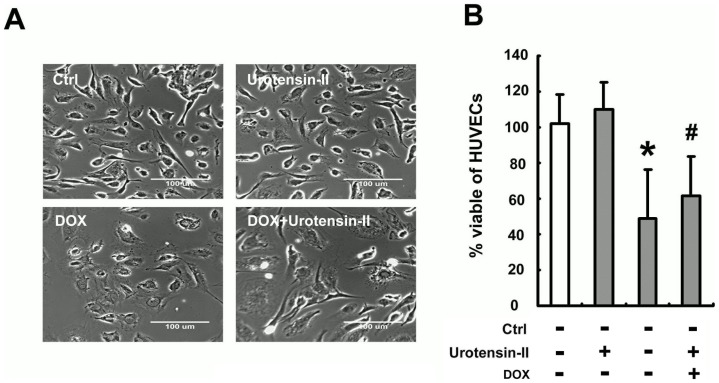
U-II protected HUVECs from DOX -induced apoptosis. (A) Photomicrographs from phase-contrast microscopy. Cell death induced by 24 h exposure to DOX (1 µM) in HUVECs replaced with fresh cell culture medium or treatment or pretreatment with U-II medium. (B) Percent cell viability using MTT assay. Densitometric data are means ± SEM from three separated experiments. *P <0.05 vs. control (Ctrl); ^#^ *P* <0.05 vs. DOX treatment.

Cell death was induced by a 24 h exposure of DOX (1 µM) in HUVECs replaced with fresh cell culture medium or treatment of U-II medium or pretreatment of U-II medium (Fig. 2A). We evaluated cell survival using MTT activity assay (Fig. 2B). Values represent means ± SEM from three separate experiments. *P <0.05 vs. untreated control; ^#^ *P* <0.05 vs. U-II pretreatment.

**Figure 3:**
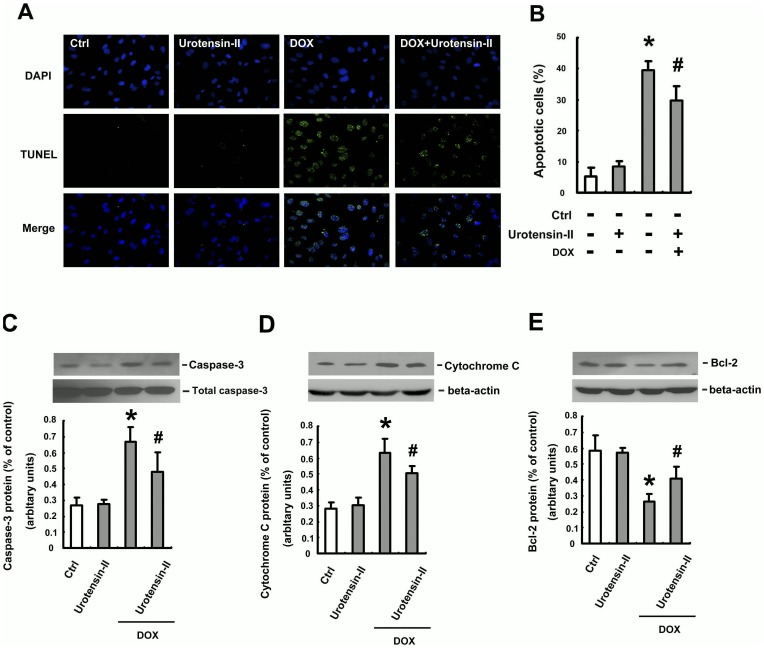
Effect of U-II on DOX-induced HUVECs apoptosis. HUVECs were incubated with DOX (1 µM) in the presence or absence of 10 nM U-II for 24 h. (A) Top: cells stained with DAPI. Middle: cells stained using TUNEL assay. Bottom: cells stained using merger. (B) Flow cytometric analysis showed that pretreatment with U-II at 10 nM significantly reduced DOX-induces cell apoptosis. The pretreatment with U-II at 10 nM significantly reduced the quantity of cleaved caspase-3 (C) and cytosol cytochrome *c* (D), compared with that in DOX-treated alone cells. (E) Cells were treated with DOX (1µM) in the presence of U-II (10 nM M) and Bcl-2 expression was upregulated. Densitometric data are means ± SEM from three separated experiments. *P <0.05 vs. control (Ctrl); ^#^ *P* <0.05 vs. DOX treatment.

The induction of apoptosis in DOX -treated HUVECs was analyzed using TUNEL and DAPI staining assays and evaluated by microscopic observation and flow cytometry. As shown in Figure , cells incubated with 1 µM DOX for 24 h showed the typical features of apoptosis, including the formation of condensed and fragmented nuclei. Those apoptotic features were not observed, however, in HUVECs pretreated with 10 nM U-II. The influence of U-II on apoptotic markers, such as cleaved caspase-3, cytochrome c and Bcl-2, was further evaluated by Western blotting analysis. The cleaved caspase-3 (Fig. 3C) and cytosol cytochrome *c* (Fig. 3D) were greatly elevated in cells treated with 1 µM of DOX for 24 h. Pretreatment with U-II at 10 nM significantly reduced the quantity of cleaved caspase-3 and cytosol cytochrome *c*, compared with that in DOX-treated alone cells. Immunoblot studies showed that U-II induced an upregulation of the anti-apoptotic Bcl-2 protein in DOX-treated cells (Fig. 3E). These results demonstrated that the pretreatment of U-II inhibited DOX-induced variation of apoptotic markers.

### U-II-mediated protective action involves PI3K/Akt and ERK activation

**Figure 4:**
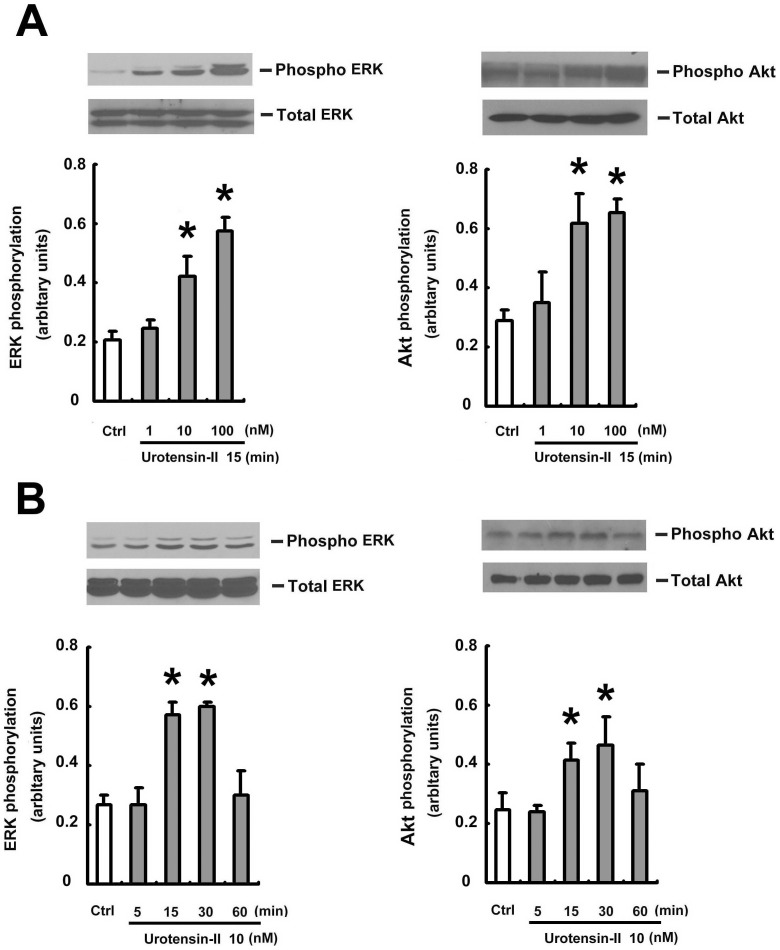
U-II induces Akt and ERK phosphorylation in HUVECs. (A) Effects of U-II on phosphorylated of Akt and ERK. HUVECs were incubated with 15 min with U-II for the indicated doses. (B) Effects of U-II on phosphorylation of ERK and Akt. HUVECs were incubated with 10 nM U-II for the indicated times. (A) and (B) Western blot analyses were performed using site- and phospho-specific ERK antibodies against Thr202/Tyr204 (p- ERK, upper blot) or total ERK (lower blot) (A). phospho-specific Akt antibodies against Ser473 (p-Akt, upper blot) or total Akt (lower blot) (B). Densitometric data are means ± SEM from three separated experiments. *P <0.05 vs. control (Ctrl).

**Figure 5:**
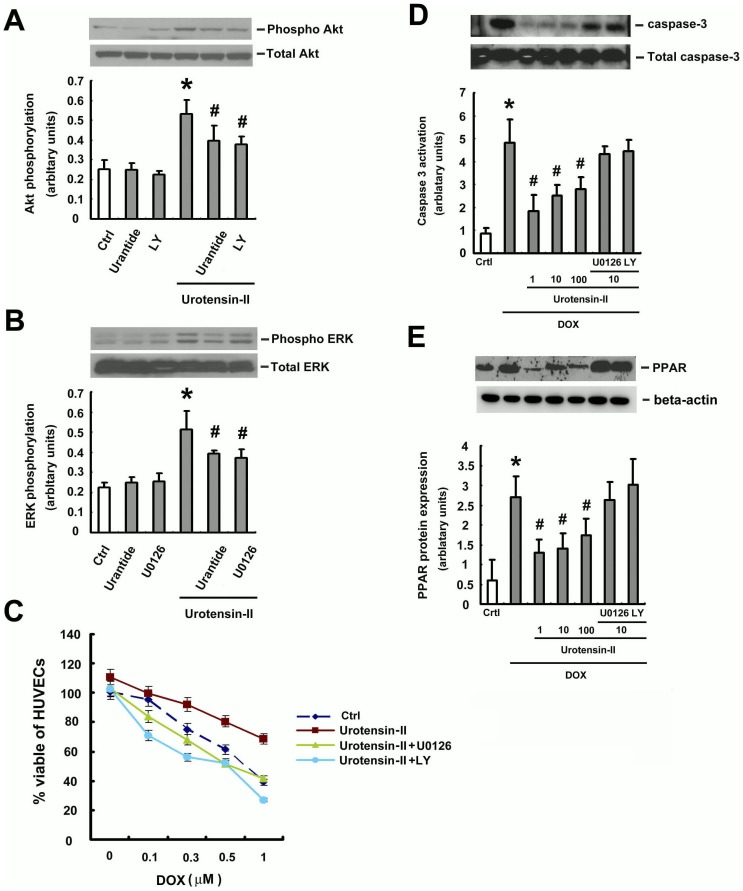
The protective effect of U-II depends on the PI3K/Akt or ERK pathway. HUVECs were pretreated with U-II antagonist urantide and PI3K/Akt inhibitor (A) and ERK inhibitor (B) 0.5 h prior to U-II treatment and then incubated for 24 h with DOX (1 µM). Viability was evaluated via western blotting analysis. (C) Treatment with LY294002 (LY) and U0126 attenuated the protective effect of U-II in DOX -treated HUVECs. Densitometric data are means ± SEM from five separated experiments. *P <0.05 vs. control; ^#^ *P* <0.05 vs. U-II only group. The protective effect of U-II was associated with cleavage of PARP and activation of caspases-3. Immunoblot studies showed that U-II induced an upregulation of activation of caspases-3 (D) and cleavage of PARP (E) in DOX-treated cells in the presence of LY294002 or U0126. Densitometric data are means ± SEM from three separated experiments. *P <0.05 vs. control (Ctrl); ^#^ *P* <0.05 vs. DOX treatment.

Activation of PI3K/Akt and ERK is well known to suppress apoptosis and promote cell survival [18], [19]. To investigate the signaling pathways activated by U-II, HUVECs were treated with U-II (1–100 nM) for increasing time periods. Phosphorylation of Akt and ERK was determined by western blot analysis. U-II increased the phosphorylation of Akt and ERK, peaking at 15 min (Fig. 4A and Fig. B). To investigate whether PI3K/Akt or ERK signaling was involved in the protective action of U-II, we pretreated cells with U-II antagonist urantide (Fig. 5A) and an ERK inhibitor (U0126) 0.5 h prior to U-II treatment (Fig. 5B) and cell toxicity determined by MTT activity assay (Fig. 5C).

In addition, the caspase family of cysteine proteases is implicated in the apoptotic process of numerous cells, and its target substrate such as PARP is proteolytically cleaved. To obtain biochemical evidence for the protective effect of UII in DOX-induced apoptosis, we investigated the caspase-3 activation and cleavage of PARP in HUVECs after PI3K inhibitor (LY294002), or an ERK inhibitor (U0126) treatment (Fig. 5E and Fig. 5D). Note that the protective effect on apoptosis is not significantly different among the 1, 10 and 100 nM of U-II (p>0.05; n = 5). The pretreatment of HUVECs with LY294002 or U0126 partially antagonized the protective action of U-II against DOX-induced cell death, indicating that PI3K/Akt or ERK activation is involved in the protective action of U-II.

### U-II inhibited induction of p53 in DOX -treated HUVEC

**Figure 6:**
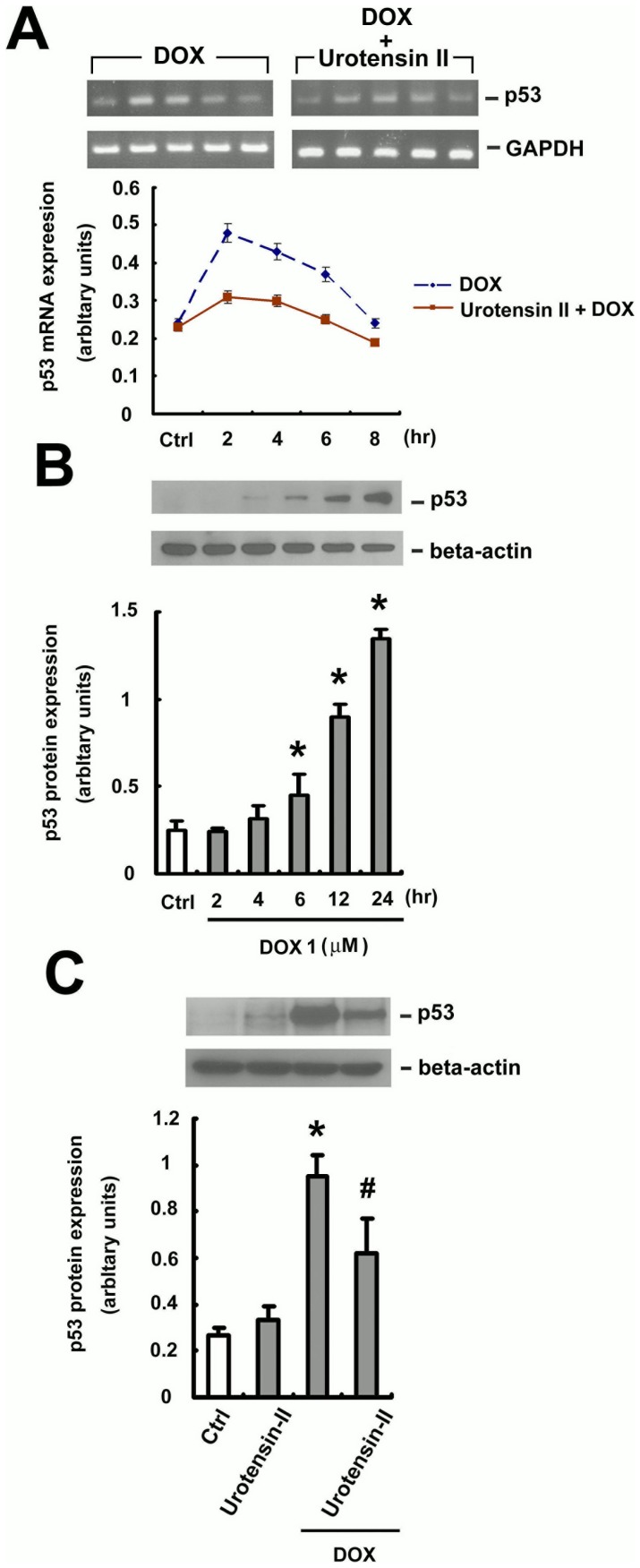
p53 expression is reduced by U-II at both mRNA and protein levels in HUVECs. (A) HUVECs were treated with DOX or DOX plus U-II. HUVECs were treated 24 h later with 1 µM DOX for the indicated time, and mRNA levels of p53 were evaluated by PCR. Equal RNA loadings were documented by GAPDH (lower). (B) HUVECs were treated with 1 µM DOX for the indicated time, and protein levels of p53 were evaluated by Western blotting. (C) HUVECs were treated with DOX or DOX plus U-II. HUVECs were treated 24 h later with or without 1 µM DOX for 24 h, and protein levels of p53 were evaluated by Western blotting. Densitometric data are means ± SEM from three separate experiments. *P <0.05 vs. control (Ctrl); ^#^ *P* <0.05 vs. DOX treatment.

DOX induced apoptosis in HUVECs via a p53-dependent mechanism [12]. Therefore, we accordingly investigated whether the protective effects of U-II against DOX-induced apoptosis occur via a p53-dependent pathway. Fig. 6A showed that p53 mRNA was rapidly induced in control cells 2 h after DOX treatment. In contrast, the induction was markedly suppressed in U-II pretreated cells. Western blot analysis showed that p53 protein of control cells accumulated in response to the application of DOX (Fig. 6B). However, the accumulation of p53 protein was significantly downregulated in U-II co-treated cells (Fig. 6C). These data clearly indicate that U-II inhibited p53 activation in DOX -treated endothelial cells at both mRNA and protein levels, suggesting that the protective effects of U-II are dependent on p53.

### U-II activated ATF3 in HUVEC

**Figure 7:**
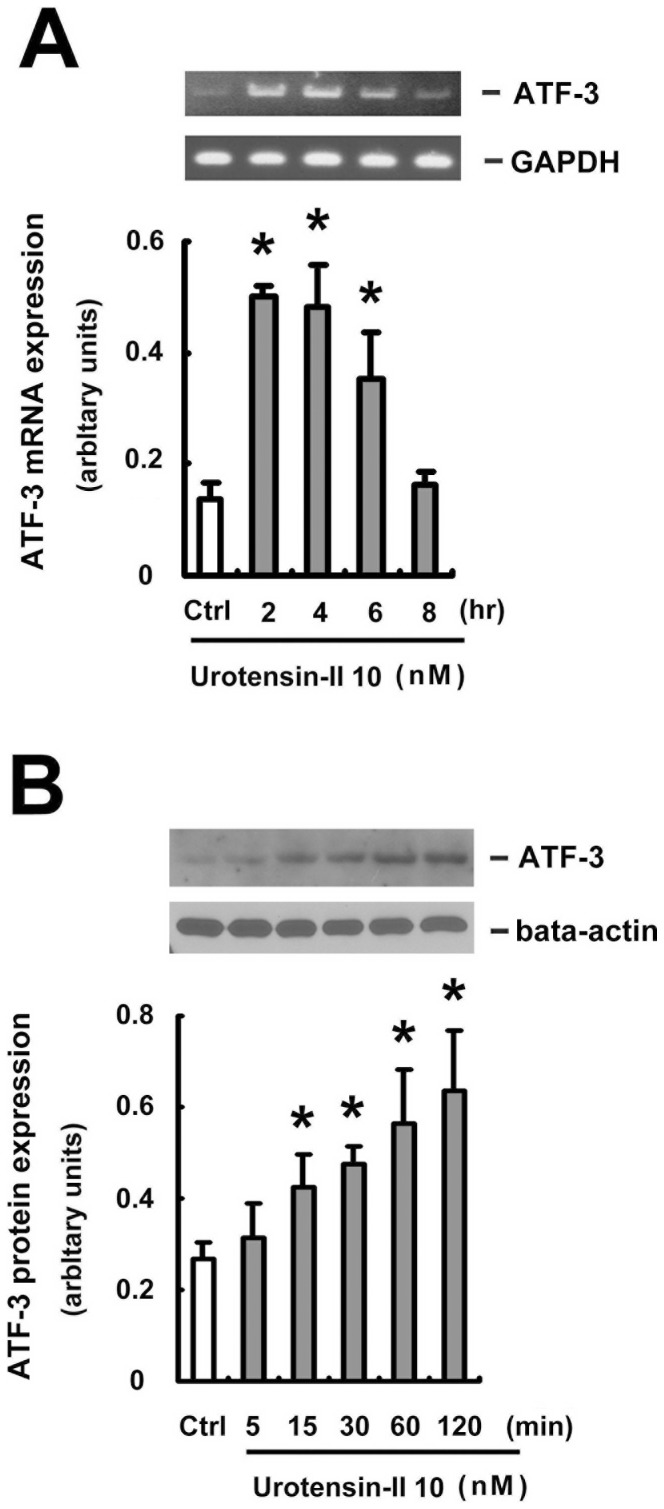
U-II activated ATF3 in HUVECs. HUVECs were treated with 10 nM U-II for the indicated time, and mRNA levels of ATF3 were evaluated by PCR. (A) Equal RNA loadings were documented by GAPDH (lower). (B) HUVECs were treated with 10 nM U-II for the indicated time, and protein levels of ATF3 were evaluated by Western blotting. Densitometric data are means ± SEM from three separate experiments. *P <0.05 vs. control (Ctrl).

Activating transcription factor 3 (ATF3) has been reported to inhibit DOX -induced apoptosis in HUVECs [20], [21]. To investigate whether U-II activates ATF3 in HUVECs, we also treated HUVECs with 10 nM U-II for increasing time periods. The mRNA levels of ATF3 were evaluated by PCR and protein levels of ATF3 were determined by Western blot analysis. As shown in Fig. 7A, ATF3 mRNA was rapidly induced 2 h after U-II treatment and then gradually returned to the basal level, compared to control cells. Fig. 7B shows that no detectable ATF3 was observed without U-II treatment. However, when the HUVECs were exposed to U-II (10 nM), ATF3 was rapidly activated 15 min after U-II treatment, and the activation continued for 60 min after stimulation. These data clearly indicate that U-II induced ATF3 activation in HUVECs at both mRNA and protein levels.

### ATF-3 was involved on U-II inhibit DOX -induced apoptosis in HUVECs

**Figure 8:**
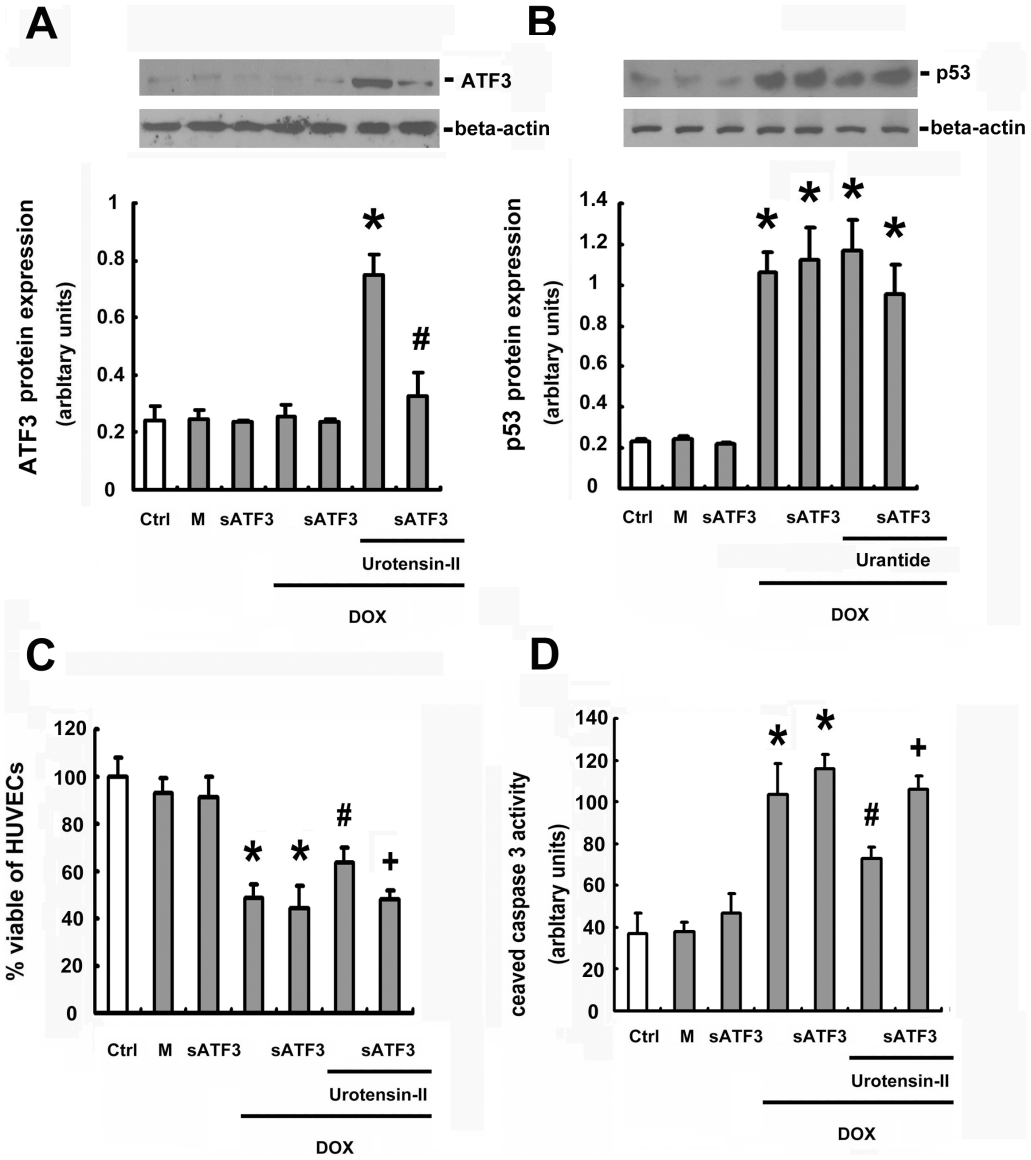
Blockage of ATF-3 attenuated the inhibitory effect of U-II on DOX -induced apoptosis. Notes: Ctrl, untransfected control; M, mock control; sATF3, ATF-3 siRNA transfection. (A) The effect of ATF-3 level when U-II regulates ATF-3 expression in DOX-treated HUVECs after ATF-3 siRNA transfection. Transfected cells were pretreated with or without U-II 10 nM for 24 h, and then treated with DOX 1 µM for 24 h. (B) The effect of p53 level when U-II regulates ATF-3 expression in DOX-treated HUVECs after ATF-3 siRNA transfection. (C) The effect of ATF-3 siRNA on U-II -decreased DOX -induced apoptosis. (D) The effect of ATF-3 siRNA on U-II -decreased DOX -induced cell viability. Transfected cells were pretreated with or without U-II 10 nM for 24 h, and then treated with DOX 1 µM for 24 h. Densitometric data are means ± SEM from three separate experiments. *p <0.05 vs the mock control, ^#^ *p* <0.05 vs DOX only group and ^+^ *p* <0.05 vs the U-II and DOX treatment.

To identify the signaling pathways involved in the effect of U-II, ATF-3 siRNA, which mitigates the expression of ATF-3, was transfected into HUVECs. The ATF-3 protein levels were noticeably reduced by ATF-3 siRNA transfection, but U-II induced ATF-3 increasing was reversed under ATF-3 siRNA transfection with DOX treatment (Fig. 8A). It has been demonstrated that TNF-κB induced p53 was downregulated by ATF-3. Therefore, we further tested the level of p53 after ATF-3 knockdown and whether ATF3-p53 regulation is inhibited by urotensin receptor antagonist. As shown in the rightmost part of Figure 8B, the DOX-induced increase of p53 was not affected when ATF-3 knockdown. Moreover, urantide (U-II antagonist) cannot reverse the effects of p53 expression under DOX treatment by ATF-3 siRNA transfection. The inhibitory effect of U-II on the DOX -induced caspase-3 activation was partially reversed by ATF-3 siRNA (Fig. 8C). Similarly, the inhibitory effect of U-II on DOX -induced HUVEC apoptosis was reduced by ATF-3 siRNA (Fig. 8D). These results revealed the involvement of the ATF-3 in U-II's effect on DOX -induced HUVEC apoptosis.

## Discussion

### U-II protect from DOX-induced apoptosis

DOX is a potent, broad-spectrum chemotherapeutic agent effective against solid tumors and malignant hematological disease. The major limiting factor for DOX is its cardiotoxicity. Several mechanisms have been suggested to underlie DOX-induced cardiotoxicity, such as enhanced ROS production, mitochondrial dysfunction, and apoptosis caused by DOX-induced DNA damage [22], [23]. A recent study demonstrates that quercetin attenuates DOX cardiotoxicity by modulating Bmi-1 expression [24]. In this study, we demonstrate that U-II-mediated protection against DOX-induced endothelial apoptosis (Fig. 1 and Fig. 2). As Bcl-2 and caspase-3 play important roles in apoptotic cell death [25], our results demonstrated that U-II down-regulates caspase-3 expression and induces Bcl-2 expression in HUVECs (Fig 3). These expression of Bcl-2 and caspase-3 is consistent with the results obtained by flow cytometry with TUNEL stain (Fig 3).

### The molecular mechanism of U-II protective action from DOX-induced impact

The mechanisms of inhibited apoptosis are associated with the activation of the cell survival-signaling cascades phosphatidylinositol 3-kinase (PI3K)/Akt or ERK [26]–[28]. The regulation of apoptosis in endothelial cells is critical for the integrity of endothelium. Studies also found that U-II promote the proliferation of rat bone marrow-derived EPCs through a process that involves ERK activation [29]. In our study, we examined the potential inhibited apoptosis function of U-II in HUVEC and the role of PI3K/Akt and ERK signaling in this process. These results demonstrated that U-II inhibits DOX-induced HUVECs death via ERK and Akt phosphorylation (see Fig. 4 and Fig. 5), and the inhibitory effect of U-II on DOX-induced apoptosis was attenuated by inhibitors of ERK (U0126) and PI3K/Akt (LY294002) (see Fig. 4 and Fig. 5).

The transcription factor p53 has been reported to play a very important role in apoptosis [23]. Activation of p53, which in turn promotes apoptosis of tumor cells, is considered to be a key mechanism of action of antitumor drugs, including DOX [24]. Numerous reports indicate that p53 tumor suppressor protein is important in regulating the apoptosis pathway, but relatively little is known about the role of p53 in apoptosis induced by DOX in vein endothelial cells. Our results showed that HUVEC treatment with DOX at 1 µM induced p53 mRNA expression for 2 h (Fig. 6A), and p53 protein activation for 6 h (Fig. 6B). This result is consistent with previous experiments showing that DOX -induced activation of p53 led to apoptosis in HUVECs [12]. Further, we found that U-II inhibited p53 activation in DOX-treated endothelial cells, suggesting that the protective effect of U-II is dependent on p53 to suppress DOX-induced cell death in HUVECs.

ATF3 is an immediate early gene found at the receiving end of multiple stress and growth stimuli. Hasin et al., reported that the AKT inhibitor (Triciribine) significantly abrogates ATF3 induction by angiotensin II in the left cardiac atrium. [30]. This report places the activation of PI3K/Akt and ERK as the up-regulators of the ATF3 inducted by angiotensin II. In our study, we revealed that U-II could induce ATF3 at both mRNA and protein levels in HUVECs (see Fig. 7A and Fig. 7B). Further, knockdown of ATF3 with ATF3 siRNA significantly reduced the U-II protective effect on DOX-induced HUVECs apoptosis (see Fig. 8A and Fig. 8B). However, whether the activation of PI3K/Akt, ERK and ATF3 inducted by U-II belong to the same signaling event or it happens in parallel needs further investigation.

### Clinical implication

Pharmacological protection of microvascular endothelium might produce benefits including reduction of myocardial toxicity and maintenance of the vascular functionality for the adequate delivery of chemotherapeutics to tumor cells. Recent study reported that U-II play an important role in vascular remodeling via the c-Src/PKC/MAPK pathway [31]. Studies also indicated that P53 is an important factor in regulating the apoptosis pathway and is associated with the DOX-induced cardiovascular injury. In our study, we found that U-II revers HUVECs viability inhibited by DOX (Fig. 1B), up-regulates ATF3 activation at both mRNA and protein levels, (Fig. 7A and Fig. 7B) and down-regulates p53 expression in HUVECs (Fig. 6A and Fig. 6B). Taken together, we anticipate that U-II could be a treatment modality in DOX-induced cardiovascular toxicity and a potential candidates to cure illnesses induced by cardiovascular remodeling, such as atherosclerosis and congestive heart failure.

In conclusion, we demonstrate that U-II has a protective effect against HUVEC damage induced by DOX via the induction of ATF3, reduction of p53 expression, and upregulation of Akt and ERK phosphorylation. This process may represent an important mechanism for the protective effect of U-II in DOX -induced apoptosis in HUVECs. These findings may shed light on the pharmacological basis of treatment of cardiovascular disease relevant to endothelial cell damage.
