# Correction: Urotensin II Inhibits Doxorubicin-Induced Human Umbilical Vein Endothelial Cell Death by Modulating ATF Expression and via the ERK and Akt Pathway

**DOI:** 10.1371/journal.pone.0118879

**Published:** 2015-03-05

**Authors:** The PLOS ONE Staff

The Data Availability statement for this paper is incorrect. The correct statement is: The authors confirm that all data underlying the findings are fully available without restriction. All relevant data are within the paper and its Supporting Information files.

Please see the Supporting Information below.

## Supporting Information

S1 Data (RAR)
